# Cognitive Aspects of Hyperactivity and Overactivity in Preadolescents with Tourette Syndrome

**DOI:** 10.1155/2013/198746

**Published:** 2013-03-10

**Authors:** Anick Laverdure, Kieron O'Connor, Marc E. Lavoie

**Affiliations:** ^1^University of Quebec in Montreal, Montreal, QC, Canada H2Y 0A3; ^2^Fernand-Seguin Research Centre, Louis-H. Lafontaine Hospital, 7331 Hochelaga Street, Montreal, QC, Canada H1N 3V2; ^3^Department of Psychoeducation and Psychology, University of Quebec in Outaouais, Gatineau, QC, Canada J8Y 3GS; ^4^Department of Psychiatry, University of Montréal, Montreal, QC, Canada H3T 1J4; ^5^Laboratoire de Psychophysiologie Cognitive et Sociale, Montreal, QC, Canada

## Abstract

Attention deficit disorder with hyperactivity (ADHD) is a common comorbidity in children with Tourette syndrome (TS). However, motor restlessness and high levels of sensorimotor activation or “overactivity” may be a feature of TS rather than a distinct ADHD comorbidity. The link between overactivity and ADHD in TS has yet to be established and in particular between adult and preadolescent manifestations. The current study furthers this understanding of ADHD features in TS by investigating the relationship between cognitive and behavioral aspects of ADHD and TS. The style of planning (STOP) overactivity scale was compared in preadolescent (*n* = 17) and adult (*n* = 17) samples. The STOP overactivity scale measures the characteristic overactive style of planning in everyday life. The aims of the study were twofold as follows: (1) to see if an overactive style was present in adolescents as well as in adults, and (2) to see if this overactive style correlated with hyperactivity, impulsivity, or perfectionism. Results suggest that overactivity may be a better description of the hyperactivity manifestations in TS. Behavioral components of overactivity were present in preadolescents while the cognitive components were more frequent in adults. Overactivity relates at the same time to perfectionism and impulsivity.

## 1. Introduction

Tourette syndrome (TS) is a tic disorder characterized by the presence of at least one phonic tic and several motor tics for 3 consecutive months (American Psychiatric Association [1]. A tic is defined as an involuntary, sudden, repetitive and stereotyped movement or vocalization. TS has to be diagnosed before the age of 18 and is more frequent in males than in females (1.5 : 1 to 3 : 1) [2–6] TS is present in 5 to 30 children and in 1 to 2 adults on 10 000 [1]. Over the last decade, many epidemiological studies have tried to establish a more representative prevalence rate of TS [7–11], and the prevalence rate of TS is estimated now to be one individual in 200 [12]. 

Rates of comorbidity vary across studies and estimates between 50% and 90% of children with TS have sufficient behavioral and emotional symptoms to meet diagnostic criteria for a comorbid disorder [13, 14]. In most cases, the externalized manifestations of these comorbid disorders may be more disruptive than the tics per se and can often be the main motivator for seeking help [12]. The disorders most frequently associated with TS are attention deficit disorder with hyperactivity (ADHD), obsessive-compulsive disorder (OCD), anxiety and mood disorders, behavioral disorders, and learning disorders [13, 15].

### 1.1. Comorbidity of Tourette Syndrome and Attention Deficit Disorder with Hyperactivity

ADHD is the disorder most often associated with TS in children. There are three types of ADHD determined by the predominant category of symptoms: inattention, hyperactivity/impulsivity, and mixed [1]. ADHD manifests in a lack of sustained attention, difficulty to concentrate, emotional instability, impulsivity, difficulties with obedience, and minor neurological signs such as deficits in fine motor coordination. ADHD is often diagnosed once the child starts school, but symptoms frequently appear during early childhood. According to the APA [1], the prevalence rate is between 3% and 7% of school-aged children, and, as with TS, ADHD is more frequent in males than in females (4 : 1 to 9 : 1). Moreover, Braswell and Bloomquist [16] have shown the cooccurrence of tic disorder in 32% of ADHD cases.

Tic disorders and ADHD occur together in at least 30–40% of cases, both in children and adults [16, 17]. Some researchers [18] have suggested that ADHD always accompanies TS, albeit sometimes at a subclinical level. The same neuronal circuits seem implicated [19], and the presence of ADHD impacts on the severity of tics and other behavioral problems. Finally, the appearance of ADHD precedes tics in 40–50% of children with TS [1], suggesting that it may be a precursor of tic onset. In adults, ADHD symptoms may lessen and adapt to align with adult functioning. O'Connor and colleagues observed the presence of a phenomenon similar to ADHD in adults with TS which they identified as “overactivity” to distinguish it from hyperactivity and which is measured by the style of planning questionnaire (STOP [20]). Recent revalidation of the STOP questionnaire [21] produced again three robust factors, accounting for 46% of the variance, involving overactivity, overpreparation and overcomplexity. The overactivity subscale seems the most specifically related to TS spectrum disorders and is linked with TS symptoms in adults. The authors define overactivity as a tendency to have difficulty to relax, not planning sufficient leisure periods, and to speak and take decisions faster than most people. At first glance, ADHD and overactivity look similar. But, apart from the absence of ADHD-like behavioral and emotional concomitants in overactivity, ADHD is associated with impulsivity and/or inattention, while overactivity in adults with TS seems more associated with perfectionism [22, 23]. Such perfectionism may translate into dissatisfaction, eagerness, and frustration regarding one's performance and a consequent tendency to try to achieve more and compulsively over prepare, which are characteristics of high-risk tic trigger situations [24]. Moreover, a high level of overactivity was observed in adults with TS without the diagnosis of ADHD, so confirming that overactivity is primarily a TS characterization [25]. These findings suggest that overactive style of planning may be a constituent of TS rather than a comorbid disorder and may be part of the heightened sensorimotor activation characteristics of TS [26].

The research questions addressed here are whether overactivity as measured by the STOP is present in preadolescents; whether overactivity can be discriminated from ADHD; whether overactivity manifests itself similarly in preadolescents and in adults with TS. We expected that: (a) preadolescent samples with TS will score in the pathological overactivity range on the STOP overactivity subscale, and (b) preadolescent scores on the overactivity subscale will show a pattern of greater positive correlations with the perfectionism scale Adaptive/Maladaptive Multidimensional Perfectionism Scale (MPS) [27] than with the hyperactivity and impulsivity measures [28, 29].

## 2. Materials and Methods

### 2.1. Preadolescents Sample

Preadolescents with TS aged between 9 and 12 were recruited from Quebec Tourette Syndrome Association (QTSA) and Fernand-Seguin Research Centre (FSRC) in Montreal.

### 2.2. Adults Sample

 A sample of comparable size was randomly extracted from pretreatment data gathered in a tic treatment program at FSRC [30].

### 2.3. Inclusion/Exclusion Criteria

The inclusion criteria for the preadolescent sample of the current study were (a) main diagnosis of TS; (b) age between 9 and 12; (c) no evidence of mental retardation; (d) no evidence of substance abuse; (e) no history or evidence of schizophrenia, bipolar disorder, or mental disorder caused by a general medical condition. However, preadolescents with ADHD, OCD, and other TS-linked comorbidity were not excluded.

### 2.4. Diagnostic Procedure

All inclusion/exclusion criteria were confirmed by a copy of a medical or psychiatric evaluation. Participants who met these criteria were included in the study. In addition, two semistructured interviews were administered; the *Tourette Syndrome Global Scale* (TSGS) [31] and the *Yale Global Tic Severity Scale *(YTGSS) [32] were completed by the parents, the teacher, and the preadolescents (evaluator). The TSGS is a complete evaluation of TS symptoms assessing tics (motor and phonic) together with subscales sensitive to social, school, and behavioral functioning. The YGTSS is the standard clinical evaluation instrument for TS and has been used in 38% of published studies on TS [24]. This instrument assesses TS manifestations on five dimensions (number, frequency, intensity, complexity, and disruption). Both TSGS and YTGSS are usually scored as global scale from 0 to 100. The TSGS assesses not only tics but behaviour, performance and motor restlessness, and dimensions germane to the current study.

The *Achenbach System of Empirically Based Assessment* (ASEBA) [29], completed by the parents, the teacher, and the preadolescents (evaluator), was used to assess six aspects according to its scales based on the diagnostic criteria of the DSM-IV, such as affective disorders, anxiety disorders, somatic disorders, attention deficit disorder with hyperactivity, oppositional/defiant disorder, and conduct disorder. The instrument possesses good psychometric properties with Cronbach alpha coefficients of internal validity between *α* = 0.73 and *α* = 0.95, excellent test-retest reliability (*r* = 0.95) and interrater agreement (*r* = 0.93 à 0.96) [33]. The short version of the *Conners* [28], completed by the parents, the teacher, and the preadolescents (evaluator), was used to assess the severity of hyperactivity symptoms during the last month. The *Conners* [28] is a self-report scale with external observer ratings of ADHD symptoms. It has 66 items and contains nine empirically derived scales that help assess a broad range of problem behaviors; inattention/memory problems: impulsivity/emotional lability; hyperactivity/restlessness; problems with Self-Concept. Internal consistency coefficients for the total sample range from 0.77 to 0.97, and 2- to 4-week test-retest reliability coefficients (Cronbach's alpha) range from 0.71 to 0.98. The *Adaptive/Maladaptive Perfectionism Scale* (AMPS) [27], completed by the preadolescents, is a 4-dimension self-administered questionnaire adapted from the *Adult Multidimensional Perfectionism Scale* (MPS) [34]. This instrument possesses a satisfactory internal consistency (*α* = 0.73–0.91) and a test-retest reliability comparable to other similar instruments [35]. The *Barratt Impulsiveness Scale* (BIS) is one of the oldest and most widely used measures of impulsive personality traits. It includes 34 items which yield subscales of attention, motor and self-control, cognitive complexity, perseverance, and cognitive instability impulsiveness. Internal consistency scores range from 0.79–0.83 for separate populations, including psychiatric patients. The *style of planning *(STOP) *questionnaire* [21, 24], completed by the preadolescents (evaluator), is a questionnaire which rates the style of planning action according to the choice of an option of overactive response or not. Items are phrased to give the person a choice between overactive and nonoveractive styles (e.g., “You have five tasks to do. Do you begin all at once or stop to reflect on the most important?"). Lower scores on the three scales of this questionnaire, overpreparation, overcomplexity, and overactivity, reflect a style of planning where the person plans to do too much (overactivity), overprepares (invests more emotional and muscular than necessary), and overcomplicates the planning (implicating unnecessary or redundant components). A lower or more negative score on the STOP indicated symptom intensity. Internal consistency coefficients (Cronbach's alpha) range from 0.51 to 0.76, and test-retest reliability coefficients range from 0.69 to 0.85. A version of the STOP overactivity subscale adapted to children (STOP-C) was used in the present study and included items such as: “are you capable to relax during 30 minutes and do nothing?,” “do you prefer to project an image as efficient?,” “do you take on too much at once?”

### 2.5. Procedure

 Before recruiting the participants, a preliminary version of the STOP-C questionnaire was administered to three preadolescents without psychiatric conditions in order to assess the feasibility of administration. In this preliminary study, the preadolescents made comments on clarity and gave improvement suggestions concerning items and responses (answering method: paper/pen versus thermometer). The items and format remained the same with wording adapted for preadolescent comprehension. The overactivity subscale validated in 2005 [24] was used in the present analysis. The purpose was to detect whether the occurrence of overactivity as identified in adults was also present in preadolescents with TS. Since the internal consistency and test-retest reliability of the adapted STOP-C version of the STOP questionnaire were not assessed, the child adapted subscale can be considered an item set.

Parents who contacted the recruitment coordinator were first assessed during a 45-minute telephone screen interview. This semistructured interview verified if their child met the inclusion criteria of the study and served to explain the procedure. Sociodemographic and developmental information were collected during this interview. Thereafter, both parents and the child met with an evaluator, a doctorate candidate specialized in children assessment and TS. After obtaining the family's consent, the evaluator assessed the child's TS symptoms severity with the TSGS and the YGTSS. The evaluator then supervised the child's completion of the Achenbach, the AMPS, and the adapted STOP questionnaires. The parents completed some questionnaires (YGTSS, Conners, and Achenbach) and with the family's consent, the teacher's questionnaires (YGTSS, Conners, and Achenbach) were forwarded to the teachers by mail.

## 3. Statistical Analysis

In order to test the first objective, adolescent and adult scores on the STOP overactivity scale were compared by Student *t*-test. The second objective was tested by Pearson product-moment correlations between measures of overactivity, perfectionism, impulsivity and hyperactivity. Effect sizes were calculated as Cohen's d [36].

## 4. Results

### 4.1. Preadolescents Sample

 The preadolescents sample (*n* = 17) consisted of 13 males and 4 females with a mean age of 10 years old (SD = 1 year). All participants had a main diagnosis of TS, 41.1% also had a diagnosis of ADHD, and one participant had OCD. The majority of participants (88.2%) were on medication. 66.7% (*n* = 10) were taking one medication; 73.3% (*n* = 11) were on psychostimulants; 46.7% (*n* = 7) were on typical or atypical antipsychotics. Clinical data is shown in Table 1.

### 4.2. Adults Sample

 A comparison group of adults with TS was selected from a population recruited through Montreal media publicity and the Fernand-Seguin Research Centre web site. A sample of 17 participants was extracted at random separately for the male and female participants who completed the evaluation process (*n* = 66). Thirteen adult male participants and four adult female participants were selected (to match for sex) as a comparison group for the preadolescent sample. The mean age of the adult participants (*n* = 17) was 37 years old (SD = 14 years). All participants had a main diagnosis of TS, two had a comorbidity of OCD, three had a specific phobia, and 35.3% were on medication. None of the adults were diagnosed with ADHD. Clinical data is given in Table 2.

### 4.3. Clinical Comparisons

Scores on the overactivity item set of the STOP indicated the presence of overactivity for both preadolescents and adults. A Pearson product-moment correlation between the Achenbach ADHD scale and the overactivity items of the STOP-C adapted for children revealed no significant relation, in preadolescents, between the ADHD scale of the Achenbach and the STOP overactivity. However, a Pearson product-moment correlation between the Conners hyperactivity scale and the overactivity scale of the STOP questionnaire adapted for preadolescents was significant (*r*(16) = −0.54, *P* < 0.05) (note: the lower the score on the overactivity scale of the STOP questionnaire and the STOP-C adapted for children, the more pathological is the result, as opposed to all the other measures).

Adults' scores on the adult perfectionism scale (MPS) were significantly higher than preadolescents' results on the AMPS. Moreover, Student's *t*-tests showed significant differences between groups on the subscales: concern over mistakes (*t*(1,29) = −2,46, *P* < 0.01, *d* = 0,91), personal standards (*t*(1,29) = −2,51, *P* < 0.01, *d* = 0,93), and compulsivity, (*t*(1,29) = 3,06, *P* < 0.005, *d* = 1,14) (doubts about actions). A Pearson product-moment correlation underlined the relation between scores on STOP overactivity items and the total perfectionism score on the AMPS in preadolescents (*r*(16) = −0.44; *P* < 0.04).

The average score of the preadolescents on the Conners impulsivity subscale was 69 (SD = 10) and corresponds to a “moderately atypical” level of impulsivity considering that the norm is 50 (SD = 10). As for the adults, their average impulsivity score of 70 (SD, 14) represents a “slightly atypical” impulsivity considering that the control norm is 62 (SD, 11) [37]. Preadolescents showed more impulsivity than adults. There was a correlation with the Conners impulsivity and STOP overactivity (*r*(16) = −0.42, *P* < 0.05) in preadolescents. In adults, there was no correlation between the BIS and STOP overactivity subscales (*r*(16) = 0.16; *P* < 0.55).

In preadolescents, the YGTSS and the TSGS total scores, which both measured the severity of TS symptoms, were not significantly correlated to STOP-C overactivity (*r*(16) = −0.12, *P* < 0.33 (preadolescent); *r*(16) = −0.01, *P* = 0.48 (preadolescent)). The YGTSS and the TSGS total scores were not significantly correlated to perfectionism as measured by the total score of the AMPS (*r*(16) = −0.03, *P* = 0.46 (preadolescent); *r*(16) = 0.002, *P* = 0.50 (preadolescent)), nor impulsivity (see Table 3).

Preadolescents had a higher average STOP overactivity score than adults, but a lower score on that instrument is interpreted as more pathological. Hence, the preadolescents showed less overactivity (i.e., lower score) than the adult comparison group. A Student's *t*-test was carried out to compare means and showed that the adults' overactivity was significantly more pathological than the preadolescents' overactivity (*t*(1,32) = 11,58, *P* < 0.001, *d* = 4,09). The large effect size indicates that overactivity differs according to participants' age.

## 5. Discussion

The main objective of the current study was to verify the presence of overactivity in preadolescents with TS. Results showed that the scores on the adapted STOP-C overactivity item set were correlated with perfectionism subscales, and although there was a correlation in preadolescents with the Conners ADHD subscale, there was no correlation with the Achenbach ADMP scale. The relationship between perfectionism and STOP-C overactivity was stronger than the relation between the STOP-C overactivity and impulsivity. Moreover, unlike the adult sample, there was no relationship between tic scale symptom severity and STOP-C overactivity or perfectionism.

The association between scores on the STOP overactivity and the Conners scale of hyperactivity was significant, but not at an exceptional level of significance. This association between hyperactivity and overactivity may be due to the wording of several items. A positive score on some behavioural items targeting motor restlessness (e.g., items such as: “I'm always on the go.” and “It's hard for me to stay in one place too long.”), on the Conners subscale, could reflect either hyperactivity or overactivity. But depending on the corresponding cognitive or motivational criteria, the same reported behaviour may be overactive or hyperactive. The positive correlation of the Conners hyperactivity scale with perfectionism supports this ambivalence behaviour of argument. O'Connor and Laverdure [25] suggest that the behavioral symptoms of overactivity, and hyperactivity are similar while the motivation behind both disorders is different. Perfectionism seems associated with overactivity whilst impulsivity and/or inattention seem rather as correlates of hyperactivity. Such a relation was observed in the preadolescent sample. However, this association between the Conners hyperactivity subscale and the STOP overactivity subscale highlights the potential for confusing the two constructs, particularly in preadolescents. 

In order to assess the association of overactivity and its cognitive component, perfectionism, found in adults with TS by O'Connor [24], the same measures were utilized in this study. Results confirmed the tendency toward a positive association between overactivity and perfectionist beliefs. This supports the hypothesis that overactivity and hyperactivity are behaviorally similar, while overactivity is linked cognitively with perfectionism. No link between overactivity and TS symptom severity was found in the current study, suggesting that tic onset and frequency, in preadolescents, may be influenced by a number of independent learned, acquired, and environmental factors besides overactive sensorimotor state.

The cognitive aspect of perfectionism may be harder to measure in preadolescents than in adults. Results seem to confirm this hypothesis, since adults had significantly higher perfectionism scores on concern over mistakes and personal standards scales, while preadolescents had a significantly higher score on the compulsivity scale of perfectionism. The highest perfectionism score in preadolescents is the more behavioral compulsivity (doubts about actions), which leads to the inference that with aging and cognitive maturity, perfectionism scores will increase. Perfectionism scores were generally higher in adults than in preadolescents. Since perfectionism seems linked with overactivity in adults, one would expect that adults' overactivity would also be more severe than in preadolescents. Preadolescents had a higher impulsivity score than adults. This supports the idea that impulsivity decreases with normal maturation, as do hyperactivity symptoms and impulsive personality traits [38].

## 6. Conclusion

This study has several limitations. The STOP-C questionnaire adapted for preadolescents has yet to be validated and should be considered an item set at present, although the items were taken from the adult validated version. The sample size used in this study is rather small, and study the requires replication on a larger sample of people with TS. 

The majority of preadolescents were on medication (82%), while this was the case for only 35% of adults. This can be partly explained by maturation and TS symptom decrease in adults, but may also have impacted it the measures completed by the parents. In fact, as noted by several authors [39–41], the parents' perception of their child's symptoms may change according to different factors, such as medication.

Despite its limits, the current study throws light on the presence of both impulsivity and inhibition in TS and suggests that both of these processes need to be considered in understanding TS symptomatology. The lack of correlation between STOP overactivity measure and tic symptomatology suggests that tic onset in preadolescents is independent of overactive style of planning. Nevertheless, behavioral manifestations of overactivity are observable in preadolescents, while the more perfectionist cognitive motivations associated with overactivity seemingly develop later in adulthood.

## Figures and Tables

**Table 1:** TS symptomatology and clinical measures in preadolescent participants.

Measure	Preadolescent		Parents		Teacher	
	*M* (SD)	*n*	*M* (SD)	*n*	*M* (SD)	*n*
TSGS	22.06 (9.59)	17	28.91 (11.72)	17	—	—
YGTSS	18.35 (6.10)	17	25.17 (10.12)	17	19.20 (9.15)	11
Achenbach—hyperactivity	60.75 (8.73)	17	66.75 (9.04)	17	56.36 (7.23)	11
Achenbach—impulsivity	58.47 (8.74)	17	67.81 (7.73)	17	55.64 (8.92)	11
Achenbach—opposition/defiance	59.41 (8.89)	17	70.04 (7.83)	17	56.36 (11.03)	11
Conners—hyperactivity	50.35 (10.78)	17	68.10 (11.98)	17	69.36 (12.53)	11
Conners—impulsivity	52.29 (8.40)	17	76.92 (11.18)	17	57.55 (17.26)	11
Conners—inattention	52.59 (10.37)	17	67.44 (11.64)	17	59.36 (12.53)	11
AMPS—total score	65.85 (5.68)	17	—	—	—	—
STOP—overactivity	28.26 (5.39)	17	—	—	—	—

**Table 2:** TS symptom and clinical measures in adults.

Measure	*M* (SD)	*n*
TSGS	31.42 (9.51)	17
YGTSS	43.50 (25.58)	17
Barratt—total score	70.35 (13.94)	17
MPS—total score	103.06 (30.44)	17
STOP—overactivity	19.79 (8.03)	17

**Table 3:** Correlation matrix of measures in preadolescents (*n* = 17).

	Hyperactivity (ASEBA)	Hyperactivity (Conners)	Impulsivity (ASEBA)	Impulsivity (Conners)	Overactivity (STOP)
Hyperactivity (ASEBA)					
Hyperactivity (Conners)	*r* = .614 *P* < .004*				
Impulsivity (ASEBA)	*r* = .727 *P* < .000*	*r* = .460 *P* < .032*			
Impulsivity (Conners)	*r* = .534 *P* < .014*	*r* = .338 *P* < .092	*r* = .762 *P* < .000*		
Overactivity (STOP)	*r* = − .228 *P* < .189	*r* = − .461 *P* < .032*	*r* = − .384 *P* < .064	*r* = − .423 *P* < .046*	
Perfectionism (ÉPA/M)	*r* = .096 *P* < .357	*r* = .517 *P* < .017*	*r* = − .115 *P* < .330	*r* = .177 *P* < .248	*r* = − .441 *P* < .038*

*Significance level (one tailed).
